# Fatal coinfection by *Mycobacterium fortuitum* and *Epicoccum* sp. with concurrent severe coccidial enteritis in an olive ridley sea turtle (*Lepidochelys olivacea*)

**DOI:** 10.1007/s11259-026-11549-x

**Published:** 2026-09-29

**Authors:** Daphne Wrobel Goldberg, Daniela Farias da Nóbrega, Ana Paula Frederico R. L. Bracarense, Igor Massahiro Souza de Suguiura, Gabriela Bezerra, Beatriz Souto Vieira, Fábio Parra Sellera, Caio Azevedo Marques, Gissandra Farias Braz, José Soares Ferreira, Marcos Bryan Hanneman

**Affiliations:** 1 Instituto Albatroz, Cabo Frio, Rio de Janeiro, Brazil; 2 Pat Animal, Laboratory of Veterinary Pathology, São José do Rio Preto, São Paulo Brazil; 3 Laboratory of Animal Pathology, Department of Preventive Veterinary Medicine, State University of Londrina, Paraná, Brazil; 4 Laboratory of Animal Immunology, Department of Immunology, Parasitology and General Pathology, State University of Londrina, Paraná, Brazil; 5 School of Veterinary Medicine, Metropolitan University of Santos, Santos, Brazil; 6 Department of Internal Medicine, School of Veterinary Medicine and Animal Science, University of São Paulo, São Paulo, Brazil; 7 Department of Preventive Veterinary Medicine and Animal Health, School of Veterinary Medicine and Animal Sciences, University of São Paulo, São Paulo, Brazil

**Keywords:** emerging pathogens, nontuberculous mycobacteria, One Health, opportunistic fungal infection, sea turtle, wildlife disease

## Abstract

Sea turtles are susceptible to opportunistic infections, particularly when debilitated, injured, or exposed to environmental stressors. Although nontuberculous mycobacteria are increasingly recognized as emerging pathogens in marine wildlife, documented cases in sea turtles remain limited, and coinfections involving mycobacteria and uncommon environmental fungi are rarely reported. Here, we describe a fatal multisystemic disease involving *Mycobacterium fortuitum* infection, *Epicoccum* sp.-associated coelomitis, and severe coccidial enteritis in an adult olive ridley sea turtle (*Lepidochelys olivacea*) stranded in Southeastern Brazil. The turtle was admitted with marked emaciation, dehydration, and lethargy and died six days after admission despite supportive care and antimicrobial therapy. Necropsy revealed multiple disseminated granulomas over the serosal surfaces of the coelomic viscera, along with pneumonia, hepatitis, cystitis, orchitis, and severe enteritis. Histopathological examination demonstrated granulocytic coelomitis with abundant intralesional septate fungal hyphae, necrosis, and aggregates of unidentified bacterial bacilli. Severe coccidial enteritis associated with intralesional bacilli was also observed. Culture and molecular analyses identified *M. fortuitum* from tissue samples, while sequencing of fungal DNA recovered from fresh and formalin-fixed tissues identified *Epicoccum* sp. These findings support a complex multisystemic disease process characterized by mycobacterial infection, opportunistic fungal coelomitis, and severe coccidial enteritis. Chronic debilitation and severe coccidial enteritis may have predisposed the turtle to systemic *M. fortuitum* infection, with *Epicoccum* sp. subsequently acting as a secondary opportunistic invader. This case reinforces the pathogenic potential of *M. fortuitum* in free-ranging sea turtles and contributes to the growing recognition of nontuberculous mycobacteria as relevant pathogens in marine wildlife. In addition, the detection of *Epicoccum* sp. expands the known host range and pathological spectrum of this rarely reported opportunistic fungus.

**Supplementary Information:**

The online version contains supplementary material available at https://doi.org/10.1007/s11259-026-11549-x.

## Background

Sea turtles are increasingly recognized as susceptible to opportunistic infections, particularly when affected by chronic debilitation, trauma, or prolonged rehabilitation (Ebani 2023; Marchiori et al. 2023; Rodriguez et al. 2023). Bacterial and fungal agents have been reported in different clinical and pathological conditions in sea turtles, but determining their pathogenic significance remains challenging because clinical signs are often nonspecific and infectious lesions may involve more than one microorganism (Domiciano et al. 2022). In addition, chronic inflammatory and granulomatous lesions may be polymicrobial (Maluta et al. 2022; Sani and Avilés-Esquivel 2024) reinforcing the importance of integrated diagnostic approaches that combine clinical evaluation, pathology, microbiology, and molecular methods (Zaika et al. 2025).

Within this broader context, nontuberculous mycobacteria have gained attention as relevant agents of chronic and systemic disease in sea turtles. *Mycobacterium* spp. are environmental organisms commonly associated with aquatic habitats and may cause granulomatous, disseminated, or neurologic disease in immunocompromised or debilitated hosts (Goldberg et al. 2023; Stacy et al. 2025). Fungi have also been reported colonizing or infecting sea turtles, especially in eggs and debilitated animals under rehabilitation; however, the prevalence and pathogenic significance of many fungal agents in sea turtles remain poorly understood (Samat et al. 2023; Umaña et al. 2025; Wang et al. 2021). *Epicoccum* spp. are dematiaceous environmental fungi commonly found in soil, plant material, and air (Ogórek and Plaskowska 2011). Although generally regarded as saprobes, they have occasional medical relevance as opportunistic organisms (Azira et al. 2016), and infections in animals are uncommon.

Herein, we report a case of fatal coinfection caused by *Mycobacterium fortuitum*, *Epicoccum* sp. and coccidian in an adult olive ridley sea turtle (*Lepidochelys olivacea*) stranded in Southeastern Brazil. The case illustrates the complexity of interpreting multiple infectious findings in a severely debilitated animal and emphasizes that detection of concurrent agents does not establish equal contributions to disease or determine their temporal sequence.

## Case presentation

### Data source

Records related to the stranding event, clinical evaluation, veterinary care, rehabilitation, and necropsy of the turtle were retrieved from the Aquatic Biota Monitoring Information System (SIMBA), the official database used by the Campos and Espírito Santo Basins Beach Monitoring Project (PMP-BC/ES) (Petrobras 2025), available at https://simba.petrobras.com.br/simba/web/sistema/. In the present study, the dataset refers exclusively to this single stranded olive ridley turtle (*L. olivacea*).

### Clinical presentation and procedures

On May 26, 2025, an adult male *L. olivacea* stranded in the city of Cabo Frio, Rio de Janeiro, Brazil, was admitted to the Rehabilitation Center of Instituto Albatroz. The animal underwent a thorough clinical evaluation that revealed marked emaciation, dehydration, and lethargy. Blood samples were collected from the dorsal cervical sinus for hematologic and biochemical analyses. Coelomic radiographs were obtained to investigate potential internal abnormalities, and additional blood samples were submitted for hemoculture. For this purpose, samples were aseptically collected into pediatric blood culture vials containing brain–heart infusion (BHI) broth. Aliquots from the vials were subsequently inoculated onto blood agar, chromogenic agar, and MacConkey agar and incubated aerobically at 37 °C for up to 7 days, with regular examination for bacterial growth.

During rehabilitation, supportive treatment focused on intravenous fluid therapy, parenteral nutritional support, and empirical antimicrobial treatment with amikacin sulfate (generic formulation; Laboratório Teuto Brasileiro S/A, Anápolis, Goiás, Brazil) at 5 mg/kg every 72 h, administered via the intravenous route after initial rehydration. Fluid replacement was based on an estimated dehydration level of 10%. For an animal weighing 33 kg, the calculated fluid deficit was 3.3 L, administered using an infusion pump at 137.5 mL/h over 24 h. Parenteral nutritional support consisted of Aminoven 10% (Fresenius Kabi, Barueri, São Paulo, Brazil) at 10 mL/kg, totaling 330 mL, and Frutovitam (Cristália Produtos Químicos Farmacêuticos Ltda., Itapira, São Paulo, Brazil) at 5 mL, diluted in 500 mL of lactated Ringer’s solution. Despite supportive care and antimicrobial treatment, the turtle died six days after admission.

### Postmortem examination

A complete necropsy was performed immediately after death following a standardized sea turtle postmortem protocol (Flint et al. 2009). Samples collected for histopathological examination included lesions from the serosal surfaces within the coelomic cavity, brain, lungs, heart, thyroid, thymus, liver, spleen, kidneys, pancreas, stomach, small intestine, colon, urinary bladder, and testes. Representative samples submitted for mycobacterial culture and molecular analyses included liver and lung fragments containing parenchyma and associated capsular or coelomic serosal granulomatous lesions, as well as separate granulomatous lesions from the coelomic cavity. These tissue compartments were not cultured separately; therefore, culture results could not determine whether *M. fortuitum* was restricted to the serosal lesions or also present within deeper parenchymal tissue. Other organs were collected for histopathology but were not submitted separately for mycobacterial culture.

### Histopathological analysis

Tissue samples collected at necropsy were fixed in 10% buffered formalin solution and processed routinely for histopathological examination. Sections were stained with hematoxylin and eosin. Ziehl–Neelsen and Fite–Faraco reactions were performed on selected tissues for detection of acid-fast bacilli, and the Periodic acid–Schiff (PAS) reaction was used for detection of fungi.

### Mycobacterial isolation and molecular identification

For mycobacterial culture, tissue samples were decontaminated with 0.75% hexadecylpyridinium chloride (HPC). After decontamination, supernatants were inoculated onto Stonebrink and Lowenstein–Jensen media and incubated at 37 °C for up to 90 days. DNA was extracted from recovered isolates using the DNeasy Blood & Tissue Kit (Qiagen, Hilden, Germany). DNA was extracted from recovered isolates using the DNeasy Blood & Tissue Kit (Qiagen, Hilden, Germany). Genus-level confirmation was performed using the single-tube multiplex PCR described by Wilton and Cousins (1992). This assay combines primers targeting mycobacterial rRNA gene sequences with primers targeting the *MPB70* gene of members of the *Mycobacterium tuberculosis* complex (MTBC). The expected amplification products are 1,030 bp for the genus *Mycobacterium*, 850 bp for *Mycobacterium intracellulare*, 372 bp for the MTBC, and 180 bp for *Mycobacterium avium*. In the present case, the multiplex PCR result was consistent with the genus *Mycobacterium* and did not produce the 372-bp MTBC-specific amplicon. Therefore, further differentiation of MTBC members by PCR amplification of regions of difference, as described by Warren et al. (2006), was not performed. Species-level identification was subsequently undertaken by sequencing the amplified mycobacterial target and phylogenetic analysis, following the molecular identification approach originally described by Telenti et al. (1993), which used PCR amplification of a mycobacterial fragment followed by restriction enzyme analysis. In the present study, sequencing and phylogenetic analysis of the amplified fragment were used instead of restriction enzyme digestion to infer the probable species identity.

### Fungal identification

Due to histological findings suggestive of fungal structures, frozen tissue fragments collected during the postmortem examination were subjected to DNA extraction (kidneys, pancreas, spleen, heart, liver, and lungs), along with formalin-fixed, paraffin-embedded (FFPE) tissues that tested positive in the histological analysis. DNA from both fresh and fixed samples was extracted according to de Souza Suguiura (2025) and subsequently used as a template for conventional PCR, employing the ITS4 and ITS5 primers to amplify the ITS1-ITS2 region, under the following thermal cycling conditions: an initial denaturation at 94 °C for 10 min, followed by 35 cycles of 94 °C for 45 s, 60 °C for 50 s, and 72 °C for 50 s, with a final extension at 72 °C for 10 min. PCR products were purified using ammonium acetate and submitted for Sanger sequencing on an ABI 3500 Genetic Analyzer using the BigDye Terminator Kit (Thermo Fisher Scientific, Waltham, MA, USA). The resulting sequences were assembled using the BioEdit software and compared with sequences available in GenBank using the Basic Local Alignment Search Tool (BLAST). The sequences obtained in this study were submitted to GenBank under the accession numbers PZ284630 (fresh spleen) and PZ284631 (FFPE coelomic lesion).

## Results

The turtle was admitted in poor body condition, with marked emaciation, dehydration, and lethargy, indicating severe systemic compromise. Hematological analysis revealed leukocytosis (17,000 leukocytes/µL), marked heterophilia (14,480 heterophils/µL), lymphopenia, and toxic leukocyte changes compared with published values for clinically healthy adult *L. olivacea* (Santoro and Meneses 2007; Ramírez-Acevedo et al. 2012; Espinoza-Romo et al. 2018; Reséndiz et al. 2019). Serum biochemical abnormalities included increased urea concentration, uric acid values that were above some published comparative ranges but within others, and elevated AST, GGT, and CK activities. These renal-associated analytes were interpreted cautiously because dehydration, prerenal azotemia, and renal tubular injury could not be distinguished on clinical pathology alone. Although blood culture yielded no microbial growth, radiographs of the coelomic cavity showed diffusely increased pulmonary radiopacity and thickening of the faveolar septa, findings compatible with an inflammatory or infectious pulmonary process. Collectively, these findings supported the presence of systemic disease before death but did not identify its etiology, highlighting the diagnostic challenges associated with chronic and polymicrobial infections in sea turtles. Complete laboratory results and published comparative ranges are presented in Supplementary Tables 2, and representative radiographic images are provided in Supplementary Fig. 1.

**Fig. 1:**
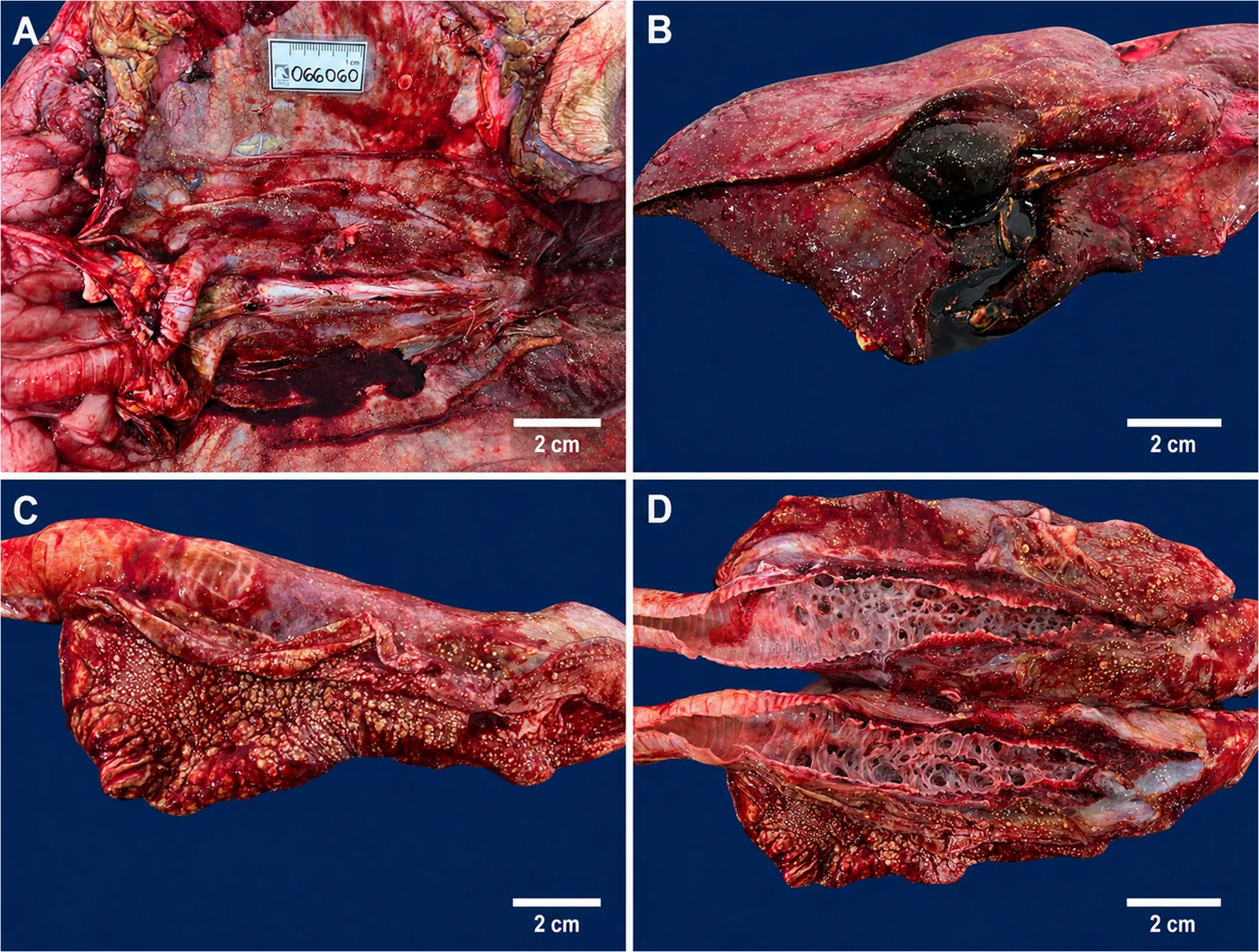
Gross granulomatous lesions in an olive ridley sea turtle (*L. olivacea*). (**A**) Multiple granulomas disseminated over the coelomic serosal surfaces. (**B**) Liver and gallbladder with granulomas on their external surfaces. (**C**) Right lung with granulomas on the coelomic surface. (**D**) Multiple granulomas within the pulmonary parenchyma. Scale bars = 2 cm

At necropsy, multiple granulomas measuring approximately 0.1 to 0.5 cm in diameter were identified. These lesions were disseminated over the serosal surfaces of the coelomic viscera, indicating extensive coelomic involvement (Fig. 1).

**Fig. 2:**
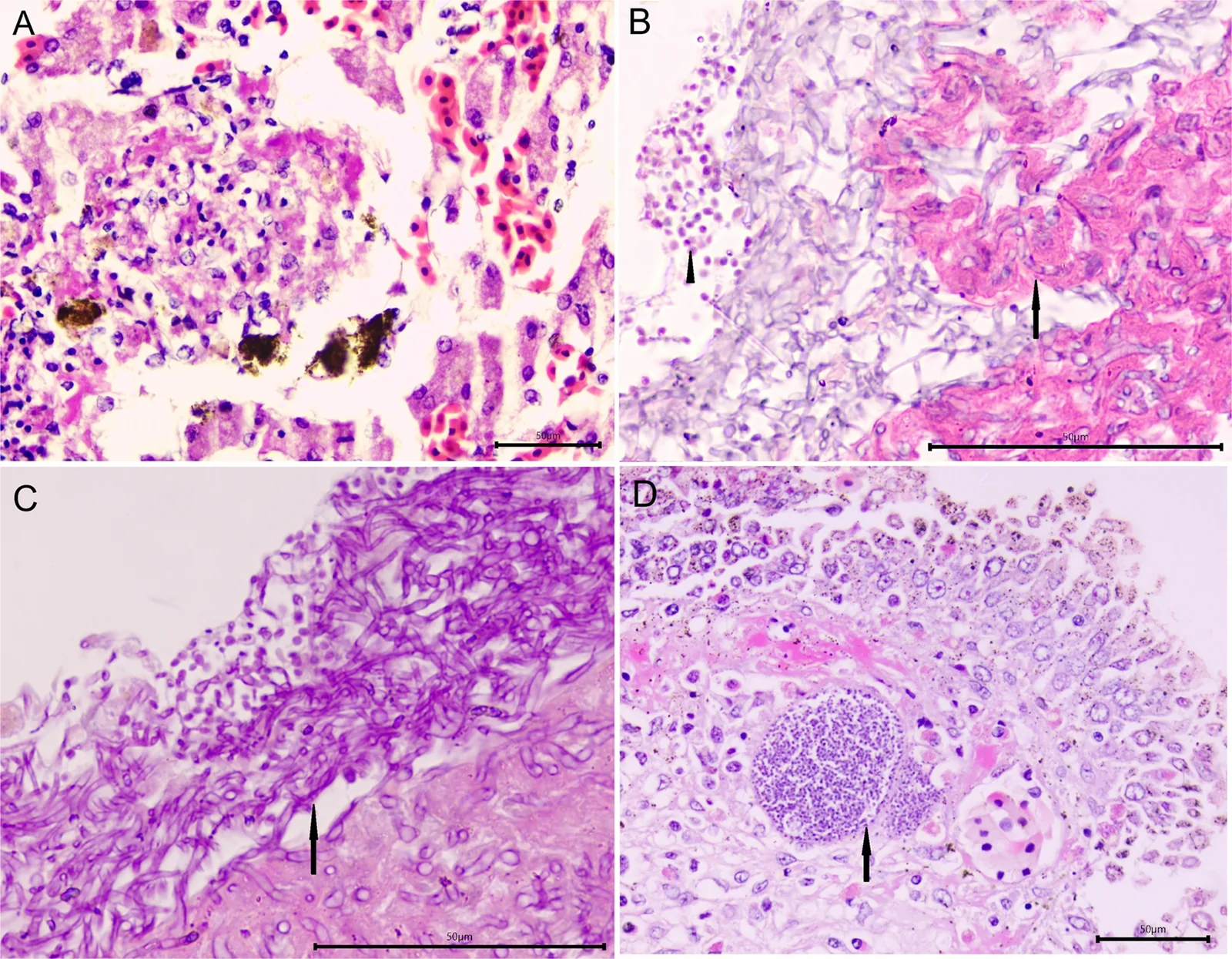
Histopathologic findings in an olive ridley sea turtle (*L. olivacea*). **(A**) Granulomatous infiltrate in the liver. HE, Obj. 40x. (**B**) Abundant small conidia (arrowhead) and weakly stained septate hyphae (arrow) in the coelomic cavity. HE, Obj. 100x. (**C**) Abundant septate hyphae (arrow) in the coelomic cavity. PAS reaction, Obj. 100x. (**D**) Schizonts of coccidia in the lamina propria of the small intestine. HE, Obj. 40x. Scale bars = 50 μm

Microscopically, the predominant lesion was granulocytic coelomitis associated with abundant intralesional fungal hyphae that were weakly stained, septate, branched, and had parallel walls, with associated small conidia, stained pink with PAS reaction (Fig. 2). Areas of necrosis were present, together with small aggregates of unidentified bacterial bacilli. Ziehl–Neelsen and Fite–Faraco reactions performed on tissue sections were negative for acid-fast bacilli. Enteritis was observed in the gastrointestinal tract. The lesion was characterized by large aggregates of oval protozoal structures morphologically consistent with coccidial schizonts within the cytoplasm of enterocytes and the lamina propria. Occasional clusters of unidentified bacilli and a marked granulocytic and lymphocytic inflammatory infiltrate were also present in the lamina propria. Occasional clusters of bacilli and a marked granulocytic and lymphocytic inflammatory infiltrate were also present in the lamina propria. Furthermore, a single coccidial schizont was observed in the hepatic parenchyma. Because this structure was not spatially associated with the granulomatous inflammatory foci, the hepatic coccidial finding was interpreted as a separate lesion rather than the cause of hepatic inflammatory changes. Additional lesions included pneumonia, colitis, cystitis, orchitis, and moderate acute renal tubular necrosis. Organ-specific findings are summarized in Supplementary Table 1.

Culture of tissue samples from the liver, lungs, and coelomic granulomatous lesions yielded *M. fortuitum*, which was confirmed by molecular assays. Because liver and lung samples included both parenchyma and associated serosal or capsular lesions and were not cultured as separate compartments, it was not possible to determine whether culture-positive mycobacteria were restricted to serosal lesions or also present within deeper parenchymal tissue.

Although acid-fast bacilli were not demonstrated histologically using Ziehl–Neelsen or Fite–Faraco staining, the sensitivity of these methods is variable, with reported ranges of 21–97% and 50–74%, respectively. This negative result may reflect a low bacterial burden, as conventional microscopy generally requires a high bacillary concentration for detection, or a focal distribution of mycobacteria within the tissue, such that the examined sections may not have contained bacilli. In addition, tissue-matrix interference and alterations of the mycobacterial cell wall caused by necrosis, autolysis, or prior antimicrobial exposure may reduce mycolic acid content and impair fuchsin retention, resulting in false-negative staining. Bacteria in a non-proliferative state may also be less readily visualized. Thus, negative histochemical staining despite a positive tissue culture is not uncommon and does not exclude mycobacterial infection (Huupponen et al. 2025; Putri et al. 2025).

The sequences obtained from fungal DNA in the analyzed tissues were successfully amplified only from the fresh spleen sample and from FFPE coelomic lesions. The sequences obtained from both samples were identical, with a complete nucleotide match. BLAST analysis indicated that both sequences belong to fungi of the *Epicoccum* genus, showing 100% similarity to the sequence of *Epicoccum* sp. (accession number: PV642725) and 99.79% similarity to other *Epicoccum* isolates, including *Epicoccum nigrum* (accession numbers: MG602595, MH645206, and MG602572) and *Epicoccum dendrobii* (OP596122 and OP596094).

Overall, the findings were consistent with systemic bacterial infection caused by *M. fortuitum*, associated opportunistic fungal infection within the coelomic cavity, severe coccidial enteritis, and inflammatory lesions containing unidentified intralesional bacilli.

## Discussion and conclusion

This report documents a fatal multisystemic disease process associated with *M. fortuitum*, *Epicoccum* sp., and severe coccidial enteritis in a debilitated adult male *L. olivacea*. The turtle was admitted with marked emaciation, dehydration, and lethargy, indicating severe systemic compromise. Clinical pathology and radiographic findings supported the presence of systemic disease before death, although they did not identify its etiology. This reinforces the diagnostic challenges associated with chronic and polymicrobial infections in sea turtles and highlights the importance of integrating clinical evaluation, diagnostic imaging, pathology, microbiological culture, and molecular methods.

Molecular and microbiological testing identified *M. fortuitum*, while fungal DNA from the spleen and a lesion within the coelomic cavity was identified as *Epicoccum* sp. Histopathological analysis demonstrated predominantly fungal and bacterial coelomitis, coccidial and bacterial enteritis, and acute-to-chronic inflammation affecting multiple organs. These concurrent findings indicate a complex multisystemic disease process but do not establish the temporal sequence or relative pathogenic contribution of each agent. In addition to the identified agents, small aggregates of unidentified bacterial bacilli were observed intralesionally in necrotic areas of the coelomic cavity and within inflamed intestinal tissue. Because these bacteria were not identified by culture or molecular methods, their taxonomic identity and pathogenic potential remain uncertain. Nevertheless, their intralesional location suggests that they may have contributed to local inflammation, tissue injury, or secondary bacterial invasion. Therefore, although they cannot be assigned a definitive primary etiological role, they should be considered part of the broader polymicrobial pathological process observed in this turtle.

From a therapeutic perspective, rapidly growing mycobacteria, including *M. fortuitum*, exhibit species- and isolate-dependent antimicrobial susceptibility, making species identification and in vitro susceptibility testing essential for selecting an effective treatment (Brown-Elliott and Woods 2019). Although amikacin was administered empirically in this case, treatment was initiated before the etiological diagnosis was established and without mycobacteria-specific susceptibility results. In addition, the advanced multisystemic nature of the disease, the poor clinical condition of the animal, and potentially limited antimicrobial penetration into granulomatous lesions may have reduced the likelihood of a favorable response. Given the short clinical course, the fatal outcome cannot be interpreted as evidence of amikacin resistance.

Moderate acute renal tubular necrosis was also observed and may have contributed to the terminal deterioration of the animal. The cause of this lesion could not be determined; however, it was likely multifactorial. Severe dehydration and hypovolemia may have resulted in renal hypoperfusion and prerenal azotemia, while systemic inflammation, sepsis, terminal shock, or toxic effects associated with bacterial or fungal products may also have contributed to acute tubular injury. Aminoglycoside-associated nephrotoxicity cannot be completely excluded because amikacin was administered during rehabilitation; however, treatment was short, was initiated after rehydration, and no drug-specific renal toxicity could be demonstrated. Therefore, the increased urea concentration and uric acid values should be interpreted cautiously, as they may reflect dehydration, renal tubular injury, or a combination of both rather than a single defined mechanism.

Recent evidence indicates that *Mycobacterium* spp. are emerging and clinically relevant pathogens in wild sea turtles, with reports documenting both novel and disseminated infections across multiple host species. In this regard, Goldberg et al. (2023) reported a disseminated infection caused by *M. gordonae* in a free-ranging juvenile green turtle (*Chelonia mydas*) from Guanabara Bay, Brazil. The turtle exhibited widespread granulomatous lesions affecting multiple organs, and intrahistiocytic acid-fast bacilli were readily observed histologically.

Conversely, the systemic presentation observed in this case differed from those reported in previous cases of mycobacterial disease in sea turtles. In a recent study, Stacy et al. (2025) described infection by a *M. ulcerans* ecovar in five wild Kemp’s ridley turtles (*Lepidochelys kempii*) and one loggerhead turtle (*Caretta caretta*). In these animals, the findings were characterized by severe meningoencephalitis, with leukocytic necrosis, fibrinoid vascular necrosis, and abundant extracellular and intrahistiocytic acid-fast bacilli, but lacked organized granuloma formation in the nervous system. Molecular characterization showed that isolates from all six turtles shared identical sequences at multiple loci, and the ITS2 sequence was identical to *M. pseudoshottsii*, supporting classification as a mycolactone-producing, *M. ulcerans*–related ecovar.

A notable feature of this case was the discrepancy between the negative Ziehl–Neelsen and Fite–Faraco staining results and the isolation of *M. fortuitum* from tissue samples, subsequently confirmed by PCR. As detailed in the Results, the absence of detectable acid-fast bacilli did not exclude infection and did not invalidate the microbiological and molecular evidence. Furthermore, the inflammatory response in the lesions within the coelomic cavity was dominated microscopically by granulocytes and abundant fungal hyphae, which may have obscured or altered lesion patterns associated with mycobacterial infection. These findings emphasize the importance of combining histopathology, culture, and molecular methods when investigating granulomatous or multisystemic disease in sea turtles.

The identification of *Epicoccum* sp. in this case is notable because this dematiaceous fungus is generally regarded as an environmental saprobe, commonly found in soil, plant material, and air, and is occasionally implicated as an opportunistic pathogen (Shenoy et al. 2012; Azira et al. 2016). Infections caused by *Epicoccum* are considered rare, particularly in animals, and its role in sea turtle pathology has not previously been documented. To our knowledge, one of the few published animal cases available in the literature is a report of *Epicoccum nigrum*–induced respiratory infection in a wild Eurasian scops owl (*Otus scops*), in which fungal infection affected the respiratory tract and pneumatic bones (Rhim et al. 2019).

Although *Epicoccum* sp. is not usually considered a primary pathogen, its detection in this case suggests opportunistic infection in a debilitated host (Portnoy et al. 1987; Azira et al. 2016). Infection by other fungi such as *Fusarium*, *Aspergillus*, *Candida*, *Cladosporium* and *Exophiala*, has been described in marine reptiles, particularly in debilitated or immunocompromised animals (Wang et al. 2021; Domiciano et al. 2022; Mastrostefano et al. 2024; Cordero-Umaña et al. 2025). Mastrostefano et al. (2024) histologically confirmed fungal infections in 20 stranded sea turtles, predominantly as severe heterophilic-to-granulomatous pneumonia associated with pulmonary granulomas. Several fungal taxa were identified, most commonly *Purpureocillium*, *Beauveria*, and *Fusarium*, demonstrating that diverse fungi should be considered in the differential diagnosis of granulomatous lesions in sea turtles. Bacterial differentials include *Nocardia* spp. and other aerobic filamentous actinomycetes, which may induce pyogranulomatous inflammation and can be mistaken histologically for fungi because of their branching filamentous morphology (Domiciano et al. 2022).

In the present case, the combination of histological findings and molecular detection of the fungus in the coelomic lesions and spleen, along with sample collection immediately after death, supports its role as a pathogen rather than an incidental environmental contaminant. Evidence of fungal infection in both the coelomic cavity and the spleen indicates an invasive profile, as the organism had to cross physiological barriers to become established in such deep tissues.

The protozoa observed in the intestine were morphologically evaluated and were consistent with coccidia; however, species identification was not possible, and molecular testing was not performed.

Importantly, the concurrent detection of *M. fortuitum*,* Epicoccum* sp., and coccidia does not imply that all three agents contributed equally to disease progression or death. One plausible sequence is that chronic debilitation and severe coccidial enteritis caused mucosal injury, nutritional impairment, and immunological compromise, thereby predisposing the turtle to systemic infection with *M. fortuitum*. *Epicoccum* sp. may subsequently have acted as a secondary opportunistic invader of pre-existing lesions or damaged tissues. In this interpretation, *M. fortuitum* and severe coccidial enteritis may have had more substantial roles in the systemic deterioration, whereas the fungus may have contributed secondarily to coelomitis. Other sequences are also possible, including primary or concurrent bacterial and parasitic disease followed by progressive debilitation. Because this report concerns a single animal examined at one point in the disease course, the order of infection and the relative contribution of each agent cannot be determined conclusively.

The broader pathological context indicates that granulomatous and multisystemic disease in sea turtles may be caused by multiple microorganisms rather than attributable to a single agent. A regional study examining granulomatous lesions in *C. mydas* from a foraging area in southern Brazil identified both bacterial and fungal pathogens within granulomas. This finding supports the idea of mixed microbial infections occurring in wild sea turtles (Domiciano et al. 2022).

From an ecological and conservation perspective, this case expands the known spectrum of infectious agents affecting sea turtles. The source of the bacterial and fungal infections could not be determined, and environmental contaminants were not assessed in this animal. Nevertheless, exposure to persistent pollutants has been associated with immune modulation and impaired health in sea turtles, potentially increasing susceptibility to opportunistic infections (Keller et al. 2006; Arienzo 2023). Consequently, the possible influence of anthropogenic environmental stressors should be investigated in future studies through direct contaminant analyses rather than inferred from the present findings. Although *L. olivacea* is not restricted to coastal habitats, individuals may move between oceanic and nearshore environments (Petitet et al. 2023). In southern Brazil, juveniles are thought to use both neritic and oceanic environments, whereas adults appear to feed predominantly in neritic waters (Maruyama et al. 2023).

In conclusion, this report documents a rare and fatal polymicrobial multisystemic disease process associated with *M. fortuitum*, *Epicoccum* sp., severe coccidial enteritis, unidentified intralesional bacilli, and moderate acute renal tubular necrosis in a debilitated olive ridley sea turtle. These findings reinforce the pathogenic potential of *M. fortuitum* in free-ranging sea turtles and support the growing recognition of nontuberculous mycobacteria as emerging pathogens in marine wildlife. The detection of *Epicoccum* sp. further expands the known host range and pathological spectrum of this opportunistic fungus. Severe coccidial enteritis was also a clinically relevant component that likely contributed to host debilitation and disease progression. A sequence involving coccidial disease and chronic debilitation, followed by *M. fortuitum* infection, secondary opportunistic invasion by *Epicoccum* sp., and additional bacterial participation by unidentified intralesional bacilli is biologically plausible but cannot be confirmed. Moderate acute renal tubular necrosis may also have contributed to terminal deterioration, although its cause could not be determined. This case emphasizes the value of integrating clinical evaluation, diagnostic imaging, pathology, microbiological culture, and molecular methods when investigating complex multisystemic infections in sea turtles.

## Supplementary information

Below is the link to the electronic supplementary material.

Supplementary Material 1

Supplementary Material 2

Supplementary Material 3

## Data Availability

No datasets were generated or analysed during the current study.
