# Supplementary material for: Fatal coinfection by Mycobacterium fortuitum and Epicoccum sp. with concurrent severe coccidial enteritis in an olive ridley sea turtle (Lepidochelys olivacea)
Source: Vet Res Commun. 2026 Sep 29;50(6):605. doi: 10.1007/s11259-026-11549-x (PMC13624016; doi:10.1007/s11259-026-11549-x)
Supplement: Supplementary file 1 [file 11259_2026_11549_MOESM1_ESM.docx]

**Supplementary material**

**Table 1.** Histopathologic findings in an olive ridley sea turtle (*L. olivacea*).

| **Organs** | **Histopathological diagnosis** |
| --- | --- |
| Coelom | Coelomitis, granulocytic, acute, focally extensive, and marked, with intralesional hyphae and bacilli. |
| Small intestine | Enteritis granulocytic and lymphocytic, chronic active, diffuse, and marked with intralesional protozoa and bacilli. |
| Liver | Hepatitis granulomatous to granulocytic, multifocal, and moderate, with focal hemorrhage, coccidia schizont and marked congestion. |
| Lung | Pneumonia interstitial, heterophilic, acute, multifocal, and mild with moderate edema, mild hemorrhage and moderate congestion. |
| Kidney | Acute tubular necrosis, multifocal, and moderate. |
| Bladder | Cystitis lymphocytic, chronic, multifocal, and mild with serositis heterophilic, multifocal, and moderate. |
| Large intestine | Colitis, lymphocytic, chronic, multifocal, and mild. |
| Testicle | Orchitis, heterophilic, acute, multifocal, and moderate. |
| Thymus | Congestion, marked. |
| Thyroid | Congestion, marked. |
| Spleen | Congestion, moderate. |
| Pancreas | Congestion, moderate. |
| Brain | Congestion, moderate. |
| Stomach | Congestion, moderate. |
