# Supplementary material for: Fatal coinfection by Mycobacterium fortuitum and Epicoccum sp. with concurrent severe coccidial enteritis in an olive ridley sea turtle (Lepidochelys olivacea)
Source: Vet Res Commun. 2026 Sep 29;50(6):605. doi: 10.1007/s11259-026-11549-x (PMC13624016; doi:10.1007/s11259-026-11549-x)
Supplement: Supplementary file 2 [file 11259_2026_11549_MOESM2_ESM.docx]

**Supplementary material**

**Table 2.** Hematological and serum biochemical results for the male olive ridley sea turtle, *Lepidochelys olivacea*, compared with published values for the species

| Parameter | Result | Unit | Published comparative values | Interpretation |
| --- | --- | --- | --- | --- |
| Hematology |  |  |  |  |
| Erythrocytes | 0.90 | ×10⁶/µL | 0.14–0.74 adult nesting females, Mexico¹; 0.12–0.41 juveniles, China² | Above published values |
| Hemoglobin | 13.0 | g/dL | 7.2–11.2 adult breeding males, Costa Rica³ | Above published values |
| Hematocrit | 42.0 | % | 23–36 adult breeding males, Costa Rica³; 24–45 adult nesting females, Mexico¹; 19–52 adult foraging turtles, Mexico⁴ | Within broader published values; above adult breeding male values |
| Mean corpuscular volume | 466.66 | fL | 364.86–1714.29 adult nesting females, Mexico¹ | Within published values |
| Mean corpuscular hemoglobin concentration | 30.95 | g/dL | 25.9–37.7 adult breeding males, Costa Rica³ | Within published values |
| Total leukocytes | 17.00 | ×10³/µL | 0.66–13.86 adult nesting females, Mexico¹; 3.75–13.00 juveniles, China² | Above published values |
| Basophils | 0.35 | ×10³/µL | 0–0.63 adult nesting females, Mexico¹; 0–0.07 juveniles, China² | Within adult female values; above juvenile values |
| Eosinophils | 0.07 | ×10³/µL | 0.09–2.63 adult nesting females, Mexico¹; 0.09–0.65 juveniles, China² | Below published values |
| Heterophils | 14.48 | ×10³/µL | 0.49–7.90 adult nesting females, Mexico¹; 2.17–8.84 juveniles, China² | Above published values |
| Lymphocytes | 1.68 | ×10³/µL | 0.07–2.91 adult nesting females, Mexico¹; 0.92–2.54 juveniles, China² | Within published values |
| Monocytes | 0.42 | ×10³/µL | 0–0.45 adult nesting females, Mexico¹; 0.40–2.31 juveniles, China² | Within published values |
| Thrombocytes | 15.00 | ×10³/µL | 6.75–19.75 juveniles, China² | Within published values |
| Blood-cell morphology | Toxic leukocytes; erythrocytes and thrombocytes morphologically normal | — | Published studies describe normal blood-cell morphology in clinically healthy turtles¹,² | Toxic leukocyte changes observed |
| Examination for hemoparasites | Not detected | — | Not consistently reported in selected published comparators | Not detected |
| Serum biochemistry |  |  |  |  |
| Aspartate aminotransferase — AST | 758.0 | U/L | 16–154 adult breeding males, Costa Rica³; 30–392 adult foraging turtles, Mexico⁴ | Above published values |
| Alkaline phosphatase — ALP | 12.7 | U/L | 9–224 adult breeding males, Costa Rica³; 12–64 adult foraging turtles, Mexico⁴ | Within published values |
| Gamma-glutamyl transferase — GGT | 2.5 | U/L | 0–1 adult foraging turtles, Mexico⁴ | Above published values |
| Creatine kinase — CK | 1,796.8 | U/L | 0–1,537 adult foraging turtles, Mexico⁴ | Above published values |
| Urea | 130.5 | mg/dL | Adult foraging turtles: BUN 44–127 mg/dL⁴; adult breeding males: urea 5.3–14.6 mmol/L, equivalent to approximately BUN 14.8–40.9 mg/dL³,⁵ | Within adult foraging published values; above adult breeding male BUN-equivalent values; interpret cautiously⁵ |
| Glucose | 360.0 | mg/dL | 46.8–88.3 adult breeding males, Costa Rica³; 62–202 adult foraging turtles, Mexico⁴ | Above published values |
| Total protein | 4.29 | g/dL | 3.1–5.0 adult breeding males, Costa Rica³; 2.8–5.9 adult foraging turtles, Mexico⁴ | Within published values |
| Albumin | 1.65 | g/dL | 0.4–0.5 adult breeding males, Costa Rica³; 0.7–2.0 adult foraging turtles, Mexico⁴ | Within broader published values; above adult breeding male values |
| Globulin | 2.64 | g/dL | 1.6–3.9 adult foraging turtles, Mexico⁴ | Within published values |
| Albumin:globulin ratio | 0.62 | — | 0.2–1.1 adult foraging turtles, Mexico⁴ | Within published values |
| Phosphorus | 10.9 | mg/dL | 6.2–11.8 adult breeding males, Costa Rica³; 6.6–16.1 adult foraging turtles, Mexico⁴ | Within published values |
| Calcium | 6.63 | mg/dL | 8.0–22.4 adult breeding males, Costa Rica³,⁶; 0–10.7 adult foraging turtles, Mexico⁴ | Within adult foraging published values; below adult breeding male values |
| Uric acid | 4.38 | mg/dL | 0.17–0.84 adult breeding males, Costa Rica³,⁷ | Above published values |
| Sodium | 150.0 | mmol/L | 121–153.5 adult breeding males, Costa Rica³ | Within published values |
| Potassium | 3.5 | mmol/L | 3.8–5.8 adult breeding males, Costa Rica³ | Below published values |
| Chloride | 96.5 | mmol/L | 103.6–124 adult breeding males, Costa Rica³ | Below published values |
| Sodium:potassium ratio | 42.8 | — | Not reported in selected published comparators | Not interpreted |

¹ Ramírez-Acevedo et al. (2012): adult nesting female *Lepidochelys olivacea* from Oaxaca, Mexico. Values are reported as minimum–maximum ranges.

² Zhang et al. (2011): juvenile *Lepidochelys olivacea* maintained in China. Values are reported as ranges and were converted, when necessary, to match the units used in the present table.

³ Santoro and Meneses (2007): adult breeding *Lepidochelys olivacea* from Costa Rica. Sex-specific male values were used where available. Values were converted to conventional clinical units when necessary: hemoglobin from g/L to g/dL; packed cell volume from L/L to %; total protein and albumin from g/L to g/dL; glucose from mmol/L to mg/dL; phosphorus from mmol/L to mg/dL; calcium from mmol/L to mg/dL; and uric acid from µmol/L to mg/dL.

⁴ Espinoza-Romo et al. (2018): adult foraging *Lepidochelys olivacea* from northern Sinaloa, Mexico. Adult ranges were used when available.

⁵ The present result was reported as urea in mg/dL. For comparison with studies reporting blood urea nitrogen — BUN — the approximate BUN-equivalent value was calculated as urea/2.14, corresponding to approximately 61.0 mg/dL. Santoro and Meneses (2007) reported urea in mmol/L; for contextual comparison, values were converted approximately to BUN-equivalent mg/dL. Interpretation should be cautious because studies differ in analyte reporting, physiological state, population, and analytical methodology.

⁶ Calcium values from Santoro and Meneses (2007) were originally reported in mmol/L and converted to mg/dL using the approximate conversion factor: mmol/L × 4.0.

⁷ Uric acid values from Santoro and Meneses (2007) were reported in µmol/L and converted to mg/dL using the approximate conversion factor: µmol/L × 0.0168.

**References**

Espinoza-Romo BA, Sainz-Hernández JC, Ley-Quiñónez CP, Hart CE, Leal-Moreno R, Aguirre AA, Zavala-Norzagaray AA (2018) Blood biochemistry of olive ridley (*Lepidochelys olivacea*) sea turtles foraging in northern Sinaloa, Mexico. PLoS One 13:e0199825. <https://doi.org/10.1371/journal.pone.0199825>

Ramírez-Acevedo LM, Martínez Blas SS, Fuentes-Mascorro G (2012) Hemogram and morphological characteristics of blood cells in the olive ridley turtle (*Lepidochelys olivacea*) of Oaxaca, Mexico. Revista Científica, FCV-LUZ 22:468–476

Santoro M, Meneses A (2007) Haematology and plasma chemistry of breeding olive ridley sea turtles (*Lepidochelys olivacea*). Veterinary Record 161:818–819. <https://doi.org/10.1136/vr.161.24.818>

Zhang FY, Li PP, Gu HX, Ye MB (2011) Hematology, morphology, and ultrastructure of blood cells of juvenile olive ridley sea turtles (*Lepidochelys olivacea*). Chelonian Conservation and Biology 10:250–256. <https://doi.org/10.2744/CCB-0890.1>
