# Supplementary material for: Fatal coinfection by Mycobacterium fortuitum and Epicoccum sp. with concurrent severe coccidial enteritis in an olive ridley sea turtle (Lepidochelys olivacea)
Source: Vet Res Commun. 2026 Sep 29;50(6):605. doi: 10.1007/s11259-026-11549-x (PMC13624016; doi:10.1007/s11259-026-11549-x)
Supplement: Supplementary file 3 [file 11259_2026_11549_MOESM3_ESM.docx]

**Figure 1**. Radiographic findings in an olive ridley sea turtle (L. olivacea) in multiple projections: (A) craniocaudal; (B) right laterolateral; and (C–D) dorsoventral views. Note the diffuse bilateral increase in pulmonary radiopacity and conspicuous thickening of the faveolar septa, resulting in a generalized reticular interstitial pulmonary pattern.
